# A multi-center, multi-organ, multi-omic prediction model for treatment-induced severe oral mucositis in nasopharyngeal carcinoma

**DOI:** 10.1007/s11547-024-01901-z

**Published:** 2024-11-21

**Authors:** Alexander James Nicol, Sai-Kit Lam, Jerry Chi Fung Ching, Victor Chi Wing Tam, Xinzhi Teng, Jiang Zhang, Francis Kar Ho Lee, Kenneth C. W. Wong, Jing Cai, Shara Wee Yee Lee

**Affiliations:** 1 Department of Health Technology and Informatics, The Hong Kong Polytechnic University, Room Y910, 9/F, Block Y, Lee Shau Kee Building, Hung Hom, Kowloon, Hong Kong China; 2 Department of Biomedical Engineering, The Hong Kong Polytechnic University, Hung Hom, Hong Kong China; 3 Department of Clinical Oncology, Queen Elizabeth Hospital, Yau Ma Tei, Hong Kong China; 4 Department of Clinical Oncology, Prince of Wales Hospital, Sha Tin, Hong Kong China; 5 The Hong Kong Polytechnic University Shenzhen Research Institute, Shenzhen, 518000 China

**Keywords:** Radiomics, Dosiomics, Oral mucositis, Toxicity, Nasopharyngeal carcinoma

## Abstract

**Purpose:**

Oral mucositis (OM) is one of the most prevalent and crippling treatment-related toxicities experienced by nasopharyngeal carcinoma (NPC) patients receiving radiotherapy (RT), posing a tremendous adverse impact on quality of life. This multi-center study aimed to develop and externally validate a multi-omic prediction model for severe OM.

**Methods:**

Four hundred and sixty-four histologically confirmed NPC patients were retrospectively recruited from two public hospitals in Hong Kong. Model development was conducted on one institution (n = 363), and the other was reserved for external validation (n = 101). Severe OM was defined as the occurrence of CTCAE grade 3 or higher OM during RT. Two predictive models were constructed: 1) conventional clinical and DVH features and 2) a multi-omic approach including clinical, radiomic and dosiomic features.

**Results:**

The multi-omic model, consisting of chemotherapy status and radiomic and dosiomic features, outperformed the conventional model in internal and external validation, achieving AUC scores of 0.67 [95% CI: (0.61, 0.73)] and 0.65 [95% CI: (0.53, 0.77)], respectively, compared to the conventional model with 0.63 [95% CI: (0.56, 0.69)] and 0.56 [95% CI: (0.44, 0.67)], respectively. In multivariate analysis, only the multi-omic model signature was significantly correlated with severe OM in external validation (p = 0.017), demonstrating the independent predictive value of the multi-omic approach.

**Conclusion:**

A multi-omic model with combined clinical, radiomic and dosiomic features achieved superior pre-treatment prediction of severe OM. Further exploration is warranted to facilitate improved clinical decision-making and enable more effective and personalized care for the prevention and management of OM in NPC patients.

## Introduction

Nasopharyngeal carcinoma (NPC) is primarily treated with radiotherapy (RT) and chemotherapy because of its close proximity to critical structures. Advances in RT techniques such as intensity-modulated RT (IMRT) have demonstrated improved locoregional control, and concurrent chemoradiotherapy has become the standard of care except for T1-2N0 disease [1, 2]. However, these treatments also result in toxicity from damage to healthy tissue. Oral mucositis (OM), referring to erythema, inflammation and ulceration occurring in the mucosal lining of the mouth and pharynx, is one of the most common and painful toxicities among head and neck cancer (HNC) patients, including those suffering from NPC [3]. A meta-analysis by Li et al. reported that 99% of NPC patients experienced radiotherapy-induced OM, and 52% experienced severe OM [4]. The pain and discomfort from this condition adversely affect patients’ quality of life, and severe cases can result in unplanned hospitalization, treatment interruption or chemotherapy dose reduction, potentially jeopardizing treatment outcomes [5, 6]. The cost of care from the provider’s perspective was also found to be associated with increasing OM severity [7]. Ongoing research on the prevention and management of OM includes investigations into various strategies, such as prophylactic oral care, the use of oral rinses, topical anesthetics and low-level laser therapy [6, 8]. Identifying patients at high risk for severe OM prior to treatment initiation is crucial for implementing personalized and targeted therapeutic strategies. Evidence suggests that the risk factors for OM are multifactorial, including demographic, treatment and tumor factors, as well as emerging evidence for genetic factors and factors identified from blood or saliva tests [9]. This undoubtedly calls for a multi-faceted analysis for effective pre-treatment identification.

Published prediction models for OM were identified in a systematic scoping review [9]. Six models predicted severe OM using conventional clinical and dose-volume-histogram (DVH) features, achieving internal validation AUCs between 0.62 and 0.81 [10–15]. Examples of clinical features included chemotherapy regimen, treatment acceleration, tumor site, BMI, sex and age. DVH parameters from organs-at-risk (OARs) including the oral cavity, oral mucosa surface and parotid glands were also included in the models. Despite promising performance scores, the lack of external validation in separate centers raises questions about model generalizability and the level of evidence. In the context of translating bench-to-bedside models, conducting a multi-center study is essential for robust validation of model generalizability. This approach will leverage multifactorial risk factors to ensure comprehensive and reliable outcomes.

Alongside standard RT workup procedures for NPC patients, a comprehensive data warehouse has been established. This contains three distinct categories of high-throughput features potentially linked to the development of severe OM. Currently, there is a lack of research utilizing all categories for the prediction of severe OM.

Radiomics, referring to the high-throughput extraction of multifarious quantitative descriptors of medical image data within a specified volume of interest (VOI), offers the potential to identify patterns that may not be apparent to the human eye [16]. Radiomic features have been used to predict toxicities, such as sensorineural hearing loss [17], hypothyroidism [18] and xerostomia [19].

Radiation dose distributions for modern techniques such as IMRT can be complex and vary between patients [20]. Dosiomics, utilizing advanced computational analysis of dose distribution patterns, was introduced by Gabrys et al. for the prediction of xerostomia in HNC patients [21]. These features go beyond conventional DVH parameters to characterize dose distribution, including spatial distribution, for better investigation of toxicity dose–response. Dosiomic features have also been utilized for the prediction of other toxicities, including dysgeusia [22] and hypothyroidism [23].

Contouromics, referring to the extraction of quantitative descriptors of complex geometric relationships between VOIs, was introduced by Lam et al. for the prediction of adaptive RT eligibility in NPC [24]. Specifically, features describing distance and angular relationships between tumor and OAR pairs were extracted. Such features characterize the relative difficulty of dose sparing between patients, which could enable better identification of high-risk patients for severe OM.

Regarding the prediction of severe OM, Dong et al. reported a radiomic model consisting of contrast-enhanced CT and T1-weighted MRI textural features extracted from the primary and neck nodal gross tumor volumes, and Agheli et al. reported a CT radiomic model extracted from the oral mucosa [25, 26]. Both studies were conducted in single-center settings and, similar to those reporting on conventional prediction models, underscored the necessity for further validation. Optimal validation would be achieved through multi-center studies using independent external validation data sourced from separate institutions.

The objective of this study was to develop and externally validate a prediction model for severe OM utilizing pre-treatment clinical, DVH, radiomic, dosiomic and contouromic features extracted from multiple OARs, adhering to stringent guidelines for comprehensive reporting. To the best of our knowledge, this model represents the first external validation of a radiomic, dosiomic or contouromic approach for predicting severe OM. This validation not only enhances the evidential basis of the model but also provides a rigorous assessment of its generalizability.

## Methodology

### Quality control and transparency

**Fig. 1:**
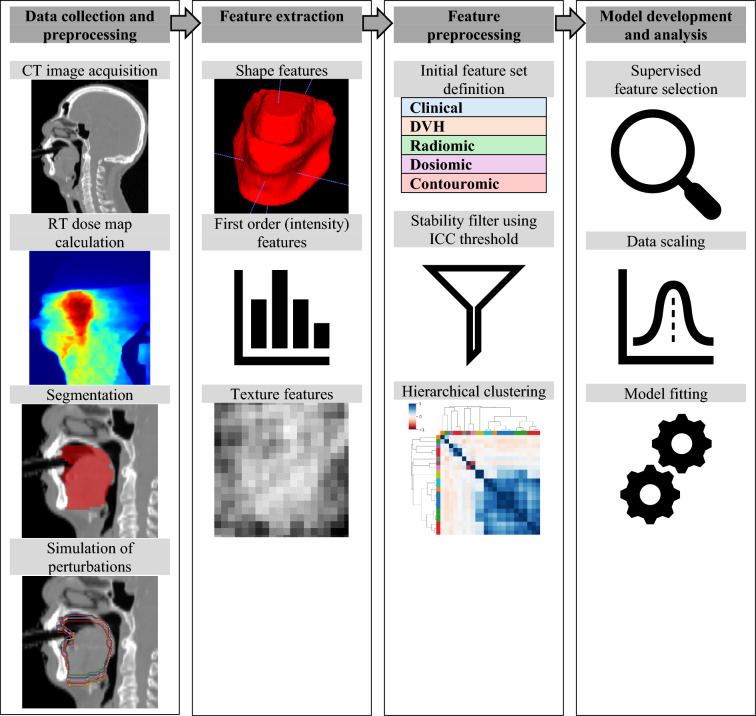
Technical workflow

This study followed the CheckList for EvaluAtion of Radiomics research (CLEAR) guidelines to ensure comprehensive reporting for more reproducible and transparent research [27]. Details of the completed checklist can be found in Appendix I. The technical workflow is shown in Fig. 1.

### Study population

Patients with histologically confirmed NPC who were treated with radiotherapy at the Queen Elizabeth Hospital (QEH), Hong Kong, between 2008 and 2018 and at the Prince of Wales Hospital (PWH), Hong Kong, between 2020 and 2021 were retrospectively enrolled in this study. Institutional review board ethics approval was obtained from each institution, and patient informed consent was waived due to the retrospective nature of the study. The exclusion criteria were: (1) patients with distant metastasis at diagnosis, (2) patients who did not have the necessary CT image or dose distribution.

**Fig. 2:**
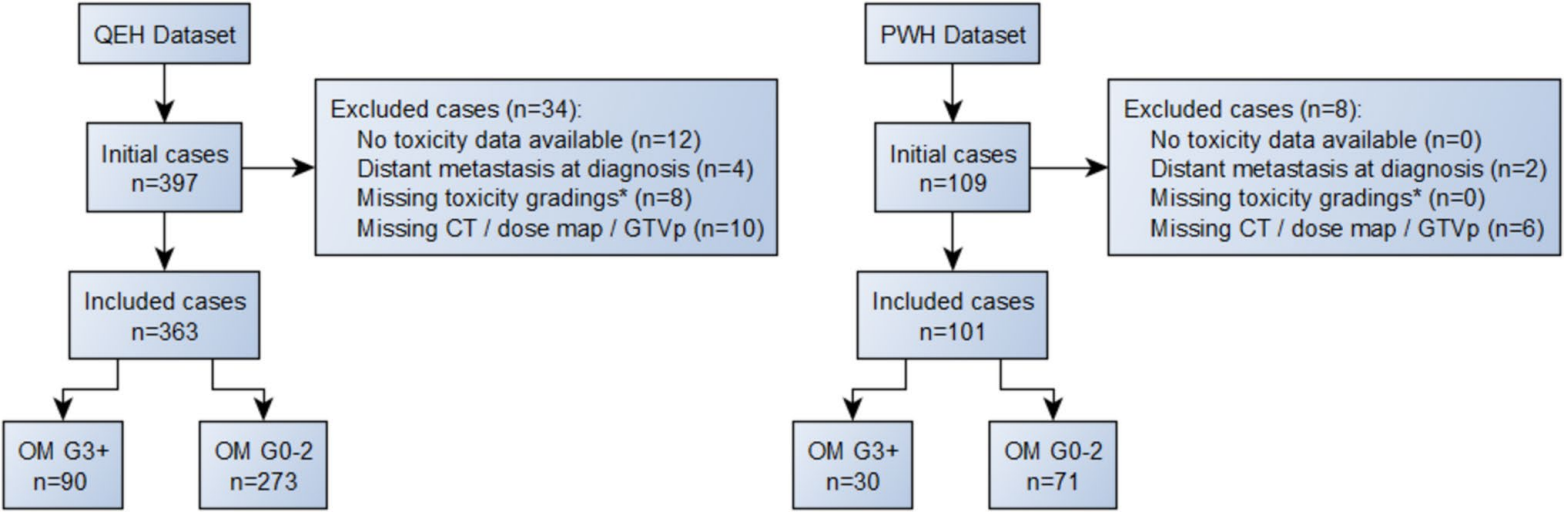
Patient recruitment diagram. *See feature data preprocessing for further details

The QEH dataset was used for model development, with the PWH dataset used for external validation. For the validation dataset, a minimum sample size of 81 patients was determined using MedCalc v22.018, to detect an AUC of 0.7 versus a null hypothesis value of 0.5 with 80% power and 0.05 significance level, assuming severe OM incidence of 40% [9, 28]. Patients were recruited consecutively by the scheduled start date of radiotherapy. The patient recruitment diagram is shown in Fig. 2.

### Imaging acquisition

The contrast-enhanced CT image used for RT planning and the resulting planned radiation dose distribution were collected for each patient. Imaging acquisition parameters are provided in Appendix A.

### Clinical data collection and outcome definition

Clinical data included age, sex, height, weight at CT simulation, TNM staging according to the 8th Edition of UICC/AJCC [29, 30], chemotherapy regimen and details of the radiotherapy delivery. The severe OM label was assigned to patients who had a maximum CTCAE grade of 3 (severe) or higher during weeks 1 to 7 of radiotherapy [31, 32]. The missing data handling strategy is reported in Appendix B.

### VOI segmentation

The extended oral cavity and pharyngeal constrictor (PC) muscles were selected as VOIs for this study. Several studies have previously investigated the extended oral cavity for predicting OM [10, 13, 15]. This VOI, as defined by the guidelines by Brouwer et al. [33], contained several areas that typically exhibit the most severe mucosal changes, including the soft palate, tongue and floor of the mouth [34]. The PC VOI, consisting of the superior, middle and inferior muscles, was frequently contoured as part of the RT planning process and included part of the mucosa at risk of severe reaction. Specifically, the hypopharyngeal mucosa was reported as the region experiencing the most severe OM after the soft palate [34]. Moreover, Tao et al. reported the radiation dose to the pharyngeal space as a significant predictor of OM [35]. The extended oral cavity and PC contours were automatically segmented using a deep learning model (see Appendix C). The primary and neck nodal GTVs (GTVp and GTVn), used for contouromic feature calculation, were segmented by clinicians during radiotherapy planning.

### Pre-processing and feature extraction

Radiomic features were extracted from the planning CT, including shape, first-order and texture features. Dosiomic features were extracted from the planned radiation dose, including first-order and texture features. Original first-order mean, median, minimum and maximum dose features were categorized as DVH features in subsequent analysis. Additional fractional volume and fractional dose DVH features were also calculated. Contouromic features were computed as in [24] for GTV-OAR pairs for both GTVp and GTVn to quantify the difficulty of dose sparing for each patient. The total number of extracted features was 2206, including the following feature types: clinical (8), DVH (126), radiomic (784), dosiomic (712), contouromic GTVp-OAR (288), contouromic GTVn-OAR (288). Details of the feature extraction settings are provided in Appendix D.

### Feature selection

Feature selection was performed in two phases. Firstly, features with low stability and high redundancy were removed in an unsupervised manner. Removal of unstable features was conducted as outlined in Appendix E. Redundant features were removed using a hierarchical clustering approach outlined in Appendix F. Secondly, supervised feature selection utilizing the severe OM outcome label was applied as part of the model pipeline, using Maximum-Relevance Minimum-Redundancy (mRMR) algorithm, implemented using the “mRMR-selection” package for Python [36].

### Model development

Two types of models were developed in this study: 1) conventional models using only clinical and DVH features and 2) multi-omic models using clinical, DVH, radiomic, dosiomic and contouromic features.

The model pipeline consisted of three steps: feature selection, scaling and model fitting. Different machine learning algorithms were investigated, including logistic regression with Ridge regression, Support Vector Machine (SVM) with linear and radial basis function kernels, Random Forest, XGBoost and Gaussian Naïve Bayes classifier.

**Fig. 3:**
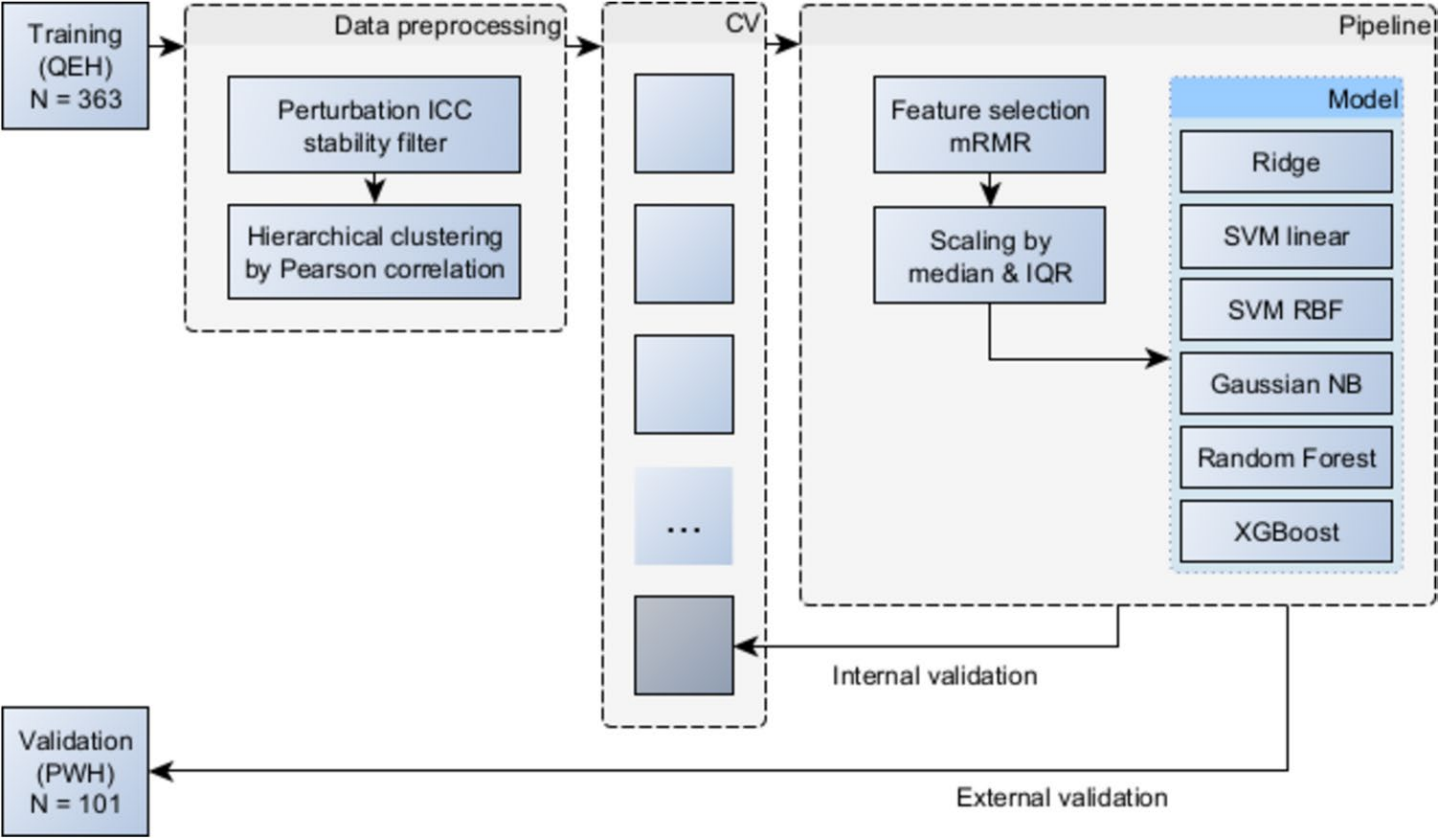
Model development flowchart. ICC = intraclass correlation coefficient, CV = cross-validation, IQR = interquartile range, SVM = support vector machine, RBF = radial basis function, NB = Naïve Bayes

The model pipeline hyperparameters, including those for mRMR and for the model, were optimized in a cross-validated grid search outlined in Fig. 3. This was conducted by maximizing the area under the receiver operating characteristic curve (AUC), a discrimination metric that is threshold-invariant and scale-invariant. Further details on the hyperparameter optimization are shown in Appendix G. The optimum settings were re-fitted on the development dataset, obtaining a training performance score for the final model which was then externally validated.

### Performance evaluation and validation

The internal validation performance was calculated from the mean AUC across the cross-validation folds and its associated 95% confidence interval (CI). CIs on the apparent (training) score and external validation scores were calculated using 1000 bootstrapped samples from QEH and PWH data, respectively.

### Feature importance assessment

Feature importance in the multi-omic model was assessed using the Shapley Additive exPlanations approach (SHAP) [37]. This method quantifies the impact of each feature on the model output.

### Decision curve analysis

The decision curve analysis was conducted for the calibrated models. The net benefit, a measure of clinical utility defined in Eq. (1), was plotted against the threshold probability (*p*_*t*_) [38]*.*

1 $$Net{ }benefit = \frac{{True{ }positives - False{ }positives \times { }\frac{{p_{t} }}{{1 - p_{t} }}}}{N}$$

## Results

### Baseline demographic and clinical characteristics

A total of 464 patients from two centers with histologically confirmed NPC were retrospectively recruited for analysis. The development dataset consisted of 363 cases from QEH, while the external validation dataset consisted of 101 cases from PWH. Baseline demographic and clinical characteristics are displayed in Table 1. Statistical significance of differences between datasets was assessed using the Mann–Whitney U test for continuous features and Fisher’s exact test for categorical features. The majority of patients were male and over 50 years old. Most patients received concurrent chemoradiotherapy, while 15% received radiotherapy alone. There were no statistically significant differences in sex, age, BMI or severe OM incidence between datasets. Differences in body weight at time of CT simulation and tumor and nodal staging reached significance level. The higher body weight for the PWH dataset may be partially explained by the higher proportion of male patients. Recruitment of patients was not stratified by tumor or nodal stage, so variation in the distribution of stages was expected.

**Table 1:** Baseline demographic and clinical characteristics. Statistically significant differences are indicated by *

Characteristic	Incidence (categorical) or median (continuous)		*P* value*
	QEH (N = 363)	PWH (N = 101)	
Male sex	268 (74%)	79 (78%)	0.419
Age at start of RT	54	57	0.358
BMI at CT simulation	23.5	24.1	0.073
Body weight at CT simulation	62.5 kg	66.7 kg	0.013*
Chemotherapy (vs RT alone)	308 (85%)	86 (85%)	1.000
Severe OM	90 (25%)	30 (30%)	0.815
T stage > = 3	315 (87%)	60 (59%)	0.012*
N stage > = 2	314 (87%)	57 (56%)	0.007*

### Conventional and multi-omic models

Conventional models with only clinical and DVH features were developed as a baseline. Multi-omic models with clinical, DVH, radiomic, dosiomic and contouromic features were also constructed. The best conventional model was a support vector machine (SVM) model with radial basis function (RBF) kernel consisting of two clinical features and two DVH features. The best multi-omic model was a Random Forest model consisting of one clinical feature, three radiomic features and one dosiomic feature. No contouromic feature was selected in the final model. None of the radiomic or dosiomic model features in the final multi-omic model were correlated (Pearson correlation coefficient > 0.5) with any clinical or DVH feature. Further details are provided in Appendix H.

**Fig. 4:**
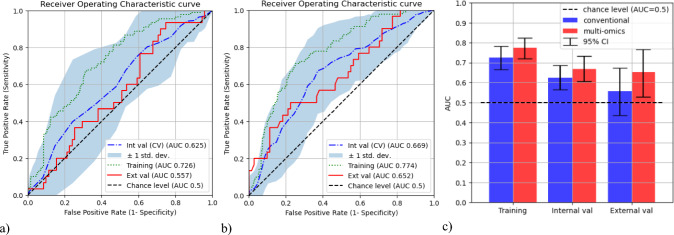
Discrimination performance of a) conventional (left), b) multi-omic (middle) models and c) comparison of AUC scores (right)

Figure 4 illustrates the enhancement in the discrimination performance from the conventional model to the multi-omic model, across training, internal validation and external validation. This improvement is evidenced by the increased AUC.

The multi-omic model outperformed the conventional model in its training, internal validation and external validation performance, achieving AUCs of 0.774 [95% CI: (0.720, 0.824)], 0.669 [95% CI: (0.606, 0.733)] and 0.652 [95% CI: (0.529, 0.767)], respectively, while the conventional model achieved AUCs of 0.725 [95% CI: (0.666, 0.782)], 0.669 [95% CI: (0.606, 0.733)] and 0.557 [95% CI: (0.436, 0.673)], respectively. The 95% confidence intervals were obtained for the training and external validation scores by generating 1000 sets of bootstrapped samples. The DeLong test did not find significant differences between the multi-omic and conventional model performance, with p values of 0.452 for the training AUCs and 0.558 for the external validation AUCs. However, it is important to note that the DeLong test is generally considered to be highly conservative [39].

The results of multivariate analysis of the conventional and multi-omic model signatures are shown in Table 2. In the development dataset, both the conventional and multi-omic models were independent predictors of severe OM. However, within the external validation dataset, only the multi-omic model demonstrated a significant association with severe OM, highlighting its unique and independent predictive power.

**Table 2:** Multivariate logistic regression of model signatures

Dataset	Variable	*P* value
Development (QEH)	Multi-omic model	0.000*
	Conventional model	0.000*
External validation (PWH)	Multi-omic model	0.017*
	Conventional model	0.742

### Decision curve analysis

**Fig. 5:**
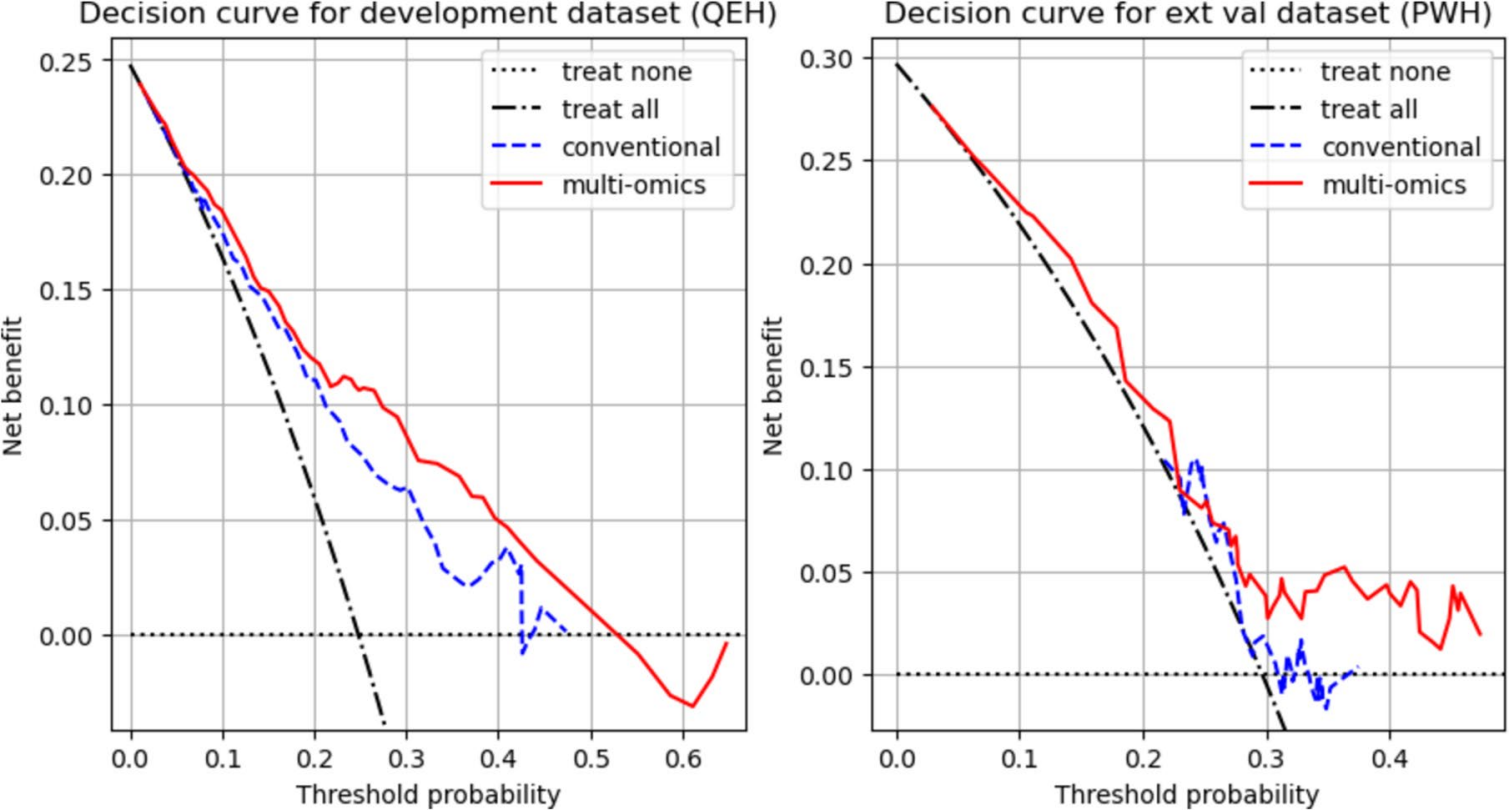
Decision curve analysis for conventional and multi-omic models in the development (left) and external validation (right) datasets

The decision curves for the conventional and multi-omic models on the development dataset (QEH) and external validation dataset (PWH) are shown in Fig. 5. In both datasets, the multi-omic model achieved a greater net benefit over the conventional model, as well as being superior to the ‘treat none’ and ‘treat all’ approaches.

### Comparison with Otter logistic model

The model developed by Otter et al. [10] achieved AUCs of 0.62 and 0.67 in internal validation within their original study and in external validation conducted by Sharabiani et al. [40]. External validation in the datasets collected for this study yielded AUCs of 0.53 and 0.66 for QEH and PWH, respectively. The data from Sharabiani and from PWH exhibited relatively high discrimination; however, the performance on the original dataset and the QEH dataset was comparatively poor. The AUC values varied widely, ranging from 0.53 to 0.67.

## Discussion

The adverse effects of OM significantly impact patient quality of life and may necessitate treatment interruptions due to complications such as pain, infection and malnutrition, often leading to hospitalization [3]. Therefore, identifying patients at high risk of severe OM early is crucial. Prompt recognition allows for timely interventions to mitigate symptoms, thereby reducing suffering and enhancing patient well-being.

This study pioneered the development of an integrated multi-omic prediction model for severe OM by uniquely combining clinical data, radiomic features and dosiomic attributes. Representing the first of its kind, this multi-omic model underwent external validation as a predictive tool for severe OM. This innovative approach not only enhances predictive accuracy but also sets a new standard for future predictive models, facilitating improved management of cancer treatment complications. With a conventional model involving only clinical and DVH features as baseline comparison, the multi-omic model outperformed the conventional model in both internal and external validations. Although the improvement in the AUC did not reach statistical significance according to the DeLong test, multivariate analysis revealed a crucial insight: The multi-omic signature was the only factor significantly associated with severe OM within the external validation dataset. Notably, the radiomic and dosiomic features integrated into the multi-omic model exhibited no correlation with clinical or DVH parameters, underscoring the model’s unique and independent predictive power. This finding emphasizes the distinctive and robust potential of the multi-omic model in predicting severe OM, independent of traditional clinical and dosimetric variables. Decision curve analysis demonstrated superior clinical utility of the multi-omic model in both datasets, underscoring the robustness of the multi-omic model as a pioneering tool in predicting severe OM. These findings strongly support further exploration of radiomics and dosiomics for predicting severe OM. This non-invasive approach significantly enhances the ability to identify patients at high risk of severe OM prior to treatment initiation, offering a crucial advancement in personalized management strategies.

Published prediction models for severe OM suffered from variable performance and a lack of external validation, with the exception of a single conventional model developed by Otter et al., [10] and validated by Sharabiani et al., [40] which consisted of only one DVH feature. The performance of this model on the QEH dataset was poor, highlighting the difficulty of developing a generalizable model across institutions. Other prediction models for severe OM have achieved high internal validation scores but lack external validation, resulting in a low level of evidence and unknown generalizability [12–15, 25].

Studies conducted by Dean, Liu and Hansen et al. have incorporated additional treatment-related variables such as choice of chemotherapeutic agents, regimen, number of treatment cycles and treatment acceleration, which may enhance the predictive value of their models [12, 13, 15]. However, it is crucial to acknowledge that treatment protocols often differ substantially between institutions and across different subtypes of HNCs, potentially affecting the generalizability of these findings.

Regarding the model features, both the conventional and multi-omic models identified chemotherapy as a critical factor increasing the risk of severe OM, aligning with prior research findings [41]. Although further investigation into the effects of specific chemotherapy drugs, the number of cycles and regimen (neoadjuvant, adjuvant or concurrent) would be beneficial; the extensive variability in chemotherapy approaches across different centers poses significant challenges in developing a generalized model. This variability highlights the complexity of creating universally applicable predictive tools in this domain. Radiomic features including the quantification of tissue texture at the back of the pharynx (PC), radiodensity within the extended oral cavity and a dosiomic texture feature indicating the spatial distribution of dose within the extended oral cavity were selected in the multi-omic model. Gabryś et al. highlighted that traditional DVH features, such as the mean dose to an organ, might not adequately characterize the risk of toxicity. They proposed that dosiomic features could offer more detailed, patient-specific and dose-independent insights [21]. The absence of contouromic features in the selection process stems from the patient geometry’s impact on severe OM being sufficiently captured through the effects incorporated within DVH and dosiomic attributes. This suggests a comprehensive representation of risk factors beyond conventional dose measures.

Despite the promising outcomes of this analysis, it is important to recognize several limitations. The severe OM label used in this study was confined to the first seven weeks from the onset of RT and did not encompass the entire 90-day period typically used to evaluate acute toxicity. However, as OM generally peaks during the fourth to fifth weeks of therapy, extending the observation period beyond seven weeks is unlikely to significantly affect the accuracy of the severe OM label [42]. This temporal boundary ensures that the critical peak of mucositis is captured, minimizing the impact of this limitation on the study’s findings. Another limitation is the exclusion of social determinants, such as smoking and alcohol consumption, due to their limited availability in the dataset. These factors have been previously identified as predictors of OM [3, 15, 43–45]. The data imbalance observed restricted the use of these features in our model development. Future studies employing multi-center cohorts should aim to construct models that more effectively generalize across the inherent structural variations between centers, thereby enhancing predictive accuracy and clinical applicability. This approach is vital for advancing the field and improving patient outcomes.

## Conclusion

In this study, a multi-omic model incorporating clinical, radiomic and dosiomic features significantly outperformed the conventional model, which relied solely on clinical and DVH features, in predicting the risk of severe OM among NPC patients undergoing RT. The multi-omic model demonstrated superior discriminatory ability not only during training but also across internal and external validation sets.

Multivariate analysis further confirmed the independent predictive power of the multi-omic model, while decision curve analysis highlighted its greater net benefit, reinforcing its clinical utility. By integrating pre-treatment assessments of tissue radiodensity and texture via radiomics, along with spatial dose distributions through dosiomics, the multi-omic model provides a comprehensive understanding of risk factors.

These findings highlight the potential of the multi-omic model to refine clinical decision-making, leading to more personalized prevention and management strategies for OM. The enhancements offered by this model underline its significant contribution to improving patient care and set a new benchmark for predictive accuracy in managing treatment-related complications in NPC patients. This study demonstrates a substantial advancement in the field, promising better patient outcomes and establishing a new standard for future predictive models.

## Appendix A: Image acquisition details

For both datasets, planning contrast-enhanced CT images were acquired with 16-slice Brilliance Big Bore CT scanners (Philips Medical Systems, Cleveland, OH). Acquisition parameters were as follows: scan mode = helical, voltage = 120 kVp, pixel spacing = 1.2 × 1.2 mm, slice thickness = 3 mm, matrix = 512 × 512px. Patients were scanned in a supine position, wearing a thermoplastic immobilization cast. Intravenous contrast agents were injected 30 s prior to scanning. In addition to the CT images, the planned radiation dose distribution and the set of contoured gross tumor volumes (GTVs) and OARs were collected.

## Appendix B: Missing data handling

The strategy for handling missing toxicity outcome data was similar to that described by Dean et al., namely that missing weekly gradings would result in the under-reporting of severe OM [46]. To mitigate this effect, patients were excluded if the maximum grade of OM was less than grade 3 (severe) and if they had less than 3 weeks of OM gradings. This resulted in eight patients being excluded from the QEH dataset and none being excluded from the PWH dataset. The three-week threshold was chosen to balance statistical power against mitigation of the under-reporting effect.

Strategies were also employed for handling missing feature data. Patients who were missing any categorical clinical features (sex, chemotherapy regimen, TNM stage) were removed. The small number of remaining missing values, such as missing BMI values, was imputed by the median feature value to minimize the impact of outliers and be suitable for various value distributions. Constant or quasi-constant continuous-valued features which were more than 20% single-valued were removed since their low variance would limit their predictive value.

## Appendix C: Auto-segmentation details

The open-source nnU-Net segmentation model architecture, a leading framework for a range of segmentation tasks in biomedicine, was used for VOI segmentation [47].

For the extended oral cavity VOI segmentation, a nnU-Net model was trained on data from 47 patients from another local hospital. Planning CT images and ground truth contours were provided for model training. The contours matched the guidelines by Brouwer [33]. Training was conducted using the default data-augmentation and cross-validation scheme provided by nnU-Net to prevent over-fitting. The trained model was then used to segment the extended oral cavity VOI for the QEH and PWH datasets.

For the PC VOI segmentation, a nnU-Net model was trained on 39 cases from the QEH dataset which had complete sets of PC contours from clinicians. Training was conducted as described above for the extended oral cavity model. The trained model was used to segment the PC VOI for the remaining patients.

**Fig. 6:**
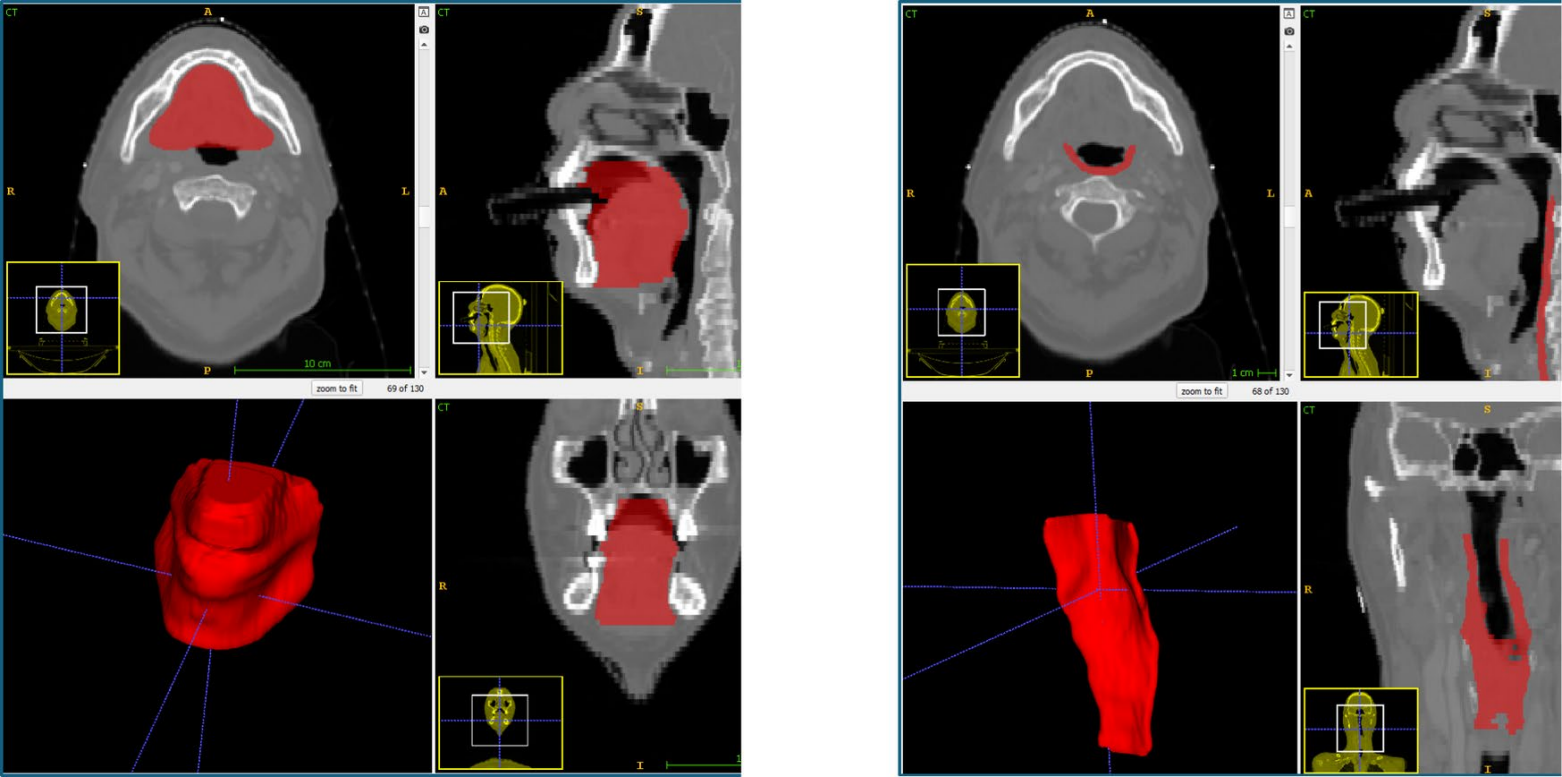
Example of extended oral cavity (extOralCavity) VOI (left) and pharyngeal constrictor (PC) VOI (right)

Examples of the extended oral cavity and PC VOIs are shown in Fig. 6.

## Appendix D: Feature extraction details

Feature extraction was compliant with a well-established protocol of the Image Biomarker Standardization Initiative (IBSI) [48]. Image pre-processing and feature extraction were performed by in-house software which utilized PyRadiomics v3.0.1 and SimpleITK v2.2.0 [49, 50]. The texture features included gray-level co-occurrence matrix (GLCM), gray-level difference matrix (GLDM), gray-level run-length matrix (GLRLM), gray-level size zone matrix (GLSZM) and neighboring gray-tone difference matrix (NGTDM) features. Features were extracted from the original and Laplacian-of-Gaussian filtered CT image and dose distribution. Aside from the specified settings, extraction settings were selected based on PyRadiomics default settings.

Contrast-enhanced planning CT images were resampled to isotropic 1 mm x 1 mm x 1 mm resolution during preprocessing. No normalization was applied to the image intensity, to preserve the physical meaning of the voxels in Hounsfield Units (HU). PyRadiomics recommends that images can be discretized such that there are between 30 and 130 bins. Therefore, CT values were discretized using a fixed bin count of 50 bins, as an intermediate value in this range. A re-segmentation range of -150 HU to 180 HU was selected to restrict the VOI to relevant soft tissues and exclude air and bone, as performed in the radiomics study by Dong et al. [25]. Features were extracted from the original CT images, as well as from Laplacian-of-Gaussian filtered images, using radius parameters of 1 mm, 2 mm and 3 mm. Laplacian-of-Gaussian filters had been previously found to produce more stable features than Wavelet filters [51] and offered additional information from their edge-detection effect.

Radiation dose distribution maps were resampled to isotropic 2.5 mm × 2.5 mm × 2.5 mm resolution to match the original pixel spacing of the dose distribution map. No normalization was applied to the intensity, whose values represent the planned dose in gray (Gy). For the dose features, a fixed bin width of 1.00 Gy was used instead of a fixed bin count, since the range of dose values would vary significantly between volumes and consequently the bin width would also vary significantly. A re-segmentation range of 0 to 100 Gy was selected to exclude any erroneous dose values. Features were extracted from the original dose map and from the Laplacian-of-Gaussian filtered dose map, using radius parameters of 1 mm, 2 mm and 3 mm.

Additional DVH features beyond those included in the intensity-based dosiomic features were calculated, including Dx_%_, the dose in Gy received by *x*% of the VOI and V*x*_Gy_, the fractional volume receiving at least *x* Gy, as defined by Gabryś et al. [21]. V*x*_%_, the fractional volume receiving at least *x*% of the maximum dose to the volume, was also calculated.

## Appendix E: Feature stability assessment using simulated perturbations

Feature stability was assessed for the development dataset (QEH) using the perturbation-based approach, outlined by Zwanenburg et al. [52]. Random translations, rotations and contour deformation using a deformation vector field (DVF) were applied using the same settings as those in the study by Zhang et al. [53]. Forty random perturbations were applied, resulting in 40 sets of perturbed features. The stability was assessed by calculating the one-way, random, absolute, single-rater intraclass correlation coefficient (ICC) for each feature. Features with poor stability against the effect of perturbations were removed using an ICC threshold of 0.7 in the development dataset.

## Appendix F: Dimensionality reduction using hierarchical clustering

A hierarchical clustering analysis was performed on the development dataset, using the Pearson correlation coefficient as the similarity metric. The feature that was conceptually and computationally simplest in each cluster was retained, in a similar approach to Gabryś et al. [21]. The first feature in each cluster of correlated features with coefficient > 0.9, was selected after sorting by ICC then by feature type, from clinical, DVH, radiomic, dosiomic to contouromic. This was performed to avoid selecting complex radiomic, dosiomic or contouromic features which could be replaced with a simpler clinical or DVH feature.

## Appendix G: Hyperparameter tuning

Feature selection, scaling and model fitting steps were incorporated into a Pipeline object from the Scikit-learn package for Python [54]. Feature selection was performed using mRMR; then, features were scaled using the RobustScaler object from the same Scikit-learn package. This method was chosen over scaling by mean and standard deviation because many features contained apparent outliers as a result of significant divergence from a normal distribution, which would distort the overall scaling [55].

A range of possible hyperparameters for the model pipeline was defined for subsequent optimization using a grid search. This set of hyperparameters is shown in Table 3. Optimization was performed by maximizing the area under the receiver operating characteristic curve (AUC), as a measure of the discrimination performance which is unaffected by class imbalance and is not dependent on setting a prediction threshold. The performance of each combination of hyperparameters was evaluated using 20-fold cross-validation. The number of folds was selected as a compromise between more conventional 5 or tenfold cross-validation and Leave One Out cross-validation. Model complexity was constrained by enforcing a minimum of 10 “events-per-variable,” in order to mitigate the risk of overfitting [56].

**Table 3:** Model hyperparameter grids

Model type	Hyperparameter grid
Ridge logistic regression	mRMR k: [1–9] C: [0.001, 0.01, 0.1, 1, 10, 100, 1000] class weight: [equal, balanced]
SVM linear	mRMR k: [1–9] C: [0.001, 0.01, 0.1, 1, 10, 100, 1000] class weight: [equal, balanced]
SVM RBF	mRMR k: [1–9] C: [0.001, 0.01, 0.1, 1, 10, 100, 1000] class weight: [equal, balanced]
Random Forest	mRMR k: [1–9] n estimators: [50] max depth: [1–9] max features: [sqrt, log2, none] class weight: [equal, balanced]
XGBoost	mRMR k: [1–9] n estimators: [50] max depth: [1–9] learning rate: [0.01, 0.1, 0.3]
Gaussian naïve bayes	mRMR k: [1–9] var smoothing: [1e-9,1e-7,1e-11]

The model pipeline, including feature selection, scaling and model fitting, was applied to each training fold, and the AUC performance on the validation fold was recorded. The best-performing hyperparameter combination was then applied and a final model was constructed using the entire development dataset. The internal validation performance was determined by the cross-validation performance for the optimal settings. Notably, this would account for the variability in the feature selection, data scaling and model optimization. Model optimization was based on the discrimination performance (AUC); however, this does not optimize the calibration of the predicted probabilities against the outcome frequencies.

## Appendix H: Results details

### Baseline characteristics

The comparison of features between severe OM groups in each dataset is shown in Table 4, along with univariate analysis. The p values were calculated using Fisher’s exact test for categorical features, and Mann–Whitney U test for continuous features. Chemotherapy and the mean dose to the GTVn were the only significant features in the development dataset. Only chemotherapy remained a significant factor in the external validation dataset.

**Table 4:** Univariate analysis of clinical and mean dose DVH features. Incidence is shown for binary features, and median value is shown for continuous features. Statistically significant differences are indicated by *

	Development (QEH)			External validation (PWH)		
Feature	Severe OM	No severe OM	*P* value	Severe OM	No severe OM	*P* value
Age at start of RT	54.5	54.0	0.180	55.5	58.0	0.132
BMI at CT simulation	23.528	23.528	0.548	24.351	23.932	0.169
Body weight at CT simulation	62.5	62.5	0.728	71.7	65.9	0.167
Chemotherapy (vs RT only)	87 (97%)	221 (81%)	< 0.001*	29 (97%)	57 (80%)	0.036*
N stage = 2	67 (74%)	200 (73%)	0.891	10 (33%)	25 (35%)	1.000
Male sex	73 (81%)	195 (71%)	0.074	24 (80%)	55 (77%)	1.000
T stage = 3	55 (61%)	187 (68%)	0.200	16 (53%)	27 (38%)	0.189
T stage = 4	21 (23%)	52 (19%)	0.368	1 (3%)	16 (23%)	0.019*
PC mean dose (Gy)	56.207	56.693	0.462	60.665	60.33	0.359
extOralCavity mean dose (Gy)	51.929	51.624	0.403	50.377	47.768	0.010*
GTVp mean dose (Gy)	73.6	73.4	0.279	72.3	72.4	0.540
GTVn mean dose (Gy)	72.4	72.1	0.018*	72.3	72.3	0.266

### Model selection

Table 5 shows the performance of different models, upon which model selection was based.

**Table 5:** Model selection. Selected models are in **bold**

Initial feature set	Model information	Ridge	SVM Linear	SVM RBF	Random Forest	XGBoost	Gaussian naïve bayes
Clinical DVH	Feature number	3	9	**4**	2	2	6
	Training AUC	0.615	0.635	**0.725**	0.689	0.692	0.666
	Internal val. (CV) AUC	0.610	0.602	**0.625**	0.607	0.609	0.582
	External val. AUC	0.543	0.601	**0.557**	0.624	0.555	0.657
Clinical DVH Radiomic Dosiomic Contouromic	Feature number	5	6	6	**5**	5	7
	Training AUC	0.707	0.699	0.725	**0.774**	0.754	0.720
	Internal val. (CV) AUC	0.664	0.668	0.648	**0.669**	0.671	0.667
	External val. AUC	0.636	0.633	0.579	**0.652**	0.603	0.591

### Model details

Tables 6 and 7 show the details of the model development for the conventional and multi-omic models. The stability of each feature, indicated by the perturbation ICC, is displayed next to each feature name.

**Table 6:** Conventional model for severe OM

Initial feature set	Clinical (N = 8) DVH (N = 126)
VOIs	Extended oral cavity PC
N features after ICC filter and hierarchical clustering	56
mRMR K	4
Model	SVM Classifier with RBF kernel, C = 10 balanced class weights
Model features	Clinical_Chemotherapy Clinical_Sex_Male PC_DVH_RelativeVolume_At_RelativeDose_0.10 (ICC = 0.87) extOralCavity_DVH_RelativeVolume_At_AbsoluteDose_70 (ICC = 0.97)

**Table 7:** Multi-omic model for severe OM

Initial feature set	Clinical (N = 8) DVH (N = 126) Radiomic (N = 784) Dosiomic (N = 712) Contouromic (N = 576)
VOIs	Extended oral cavity PC
N features after ICC filter and hierarchical clustering	507
mRMR K	5
Model	Random Forest Classifier balanced class weights max depth = 2 n_estimators = 50
Model features	Clinical_Chemotherapy PC_radiomic_original_glszm_ZoneEntropy (ICC = 0.85) extOralCavity_radiomic_log-sigma-2mm_firstorder_Median (ICC = 0.94) extOralCavity_dosiomic_original_glcm_MaximumProbability (ICC = 0.95) PC_radiomic_original_glcm_Autocorrelation (ICC = 0.76)

### Feature importance analysis

**Fig. 7:**
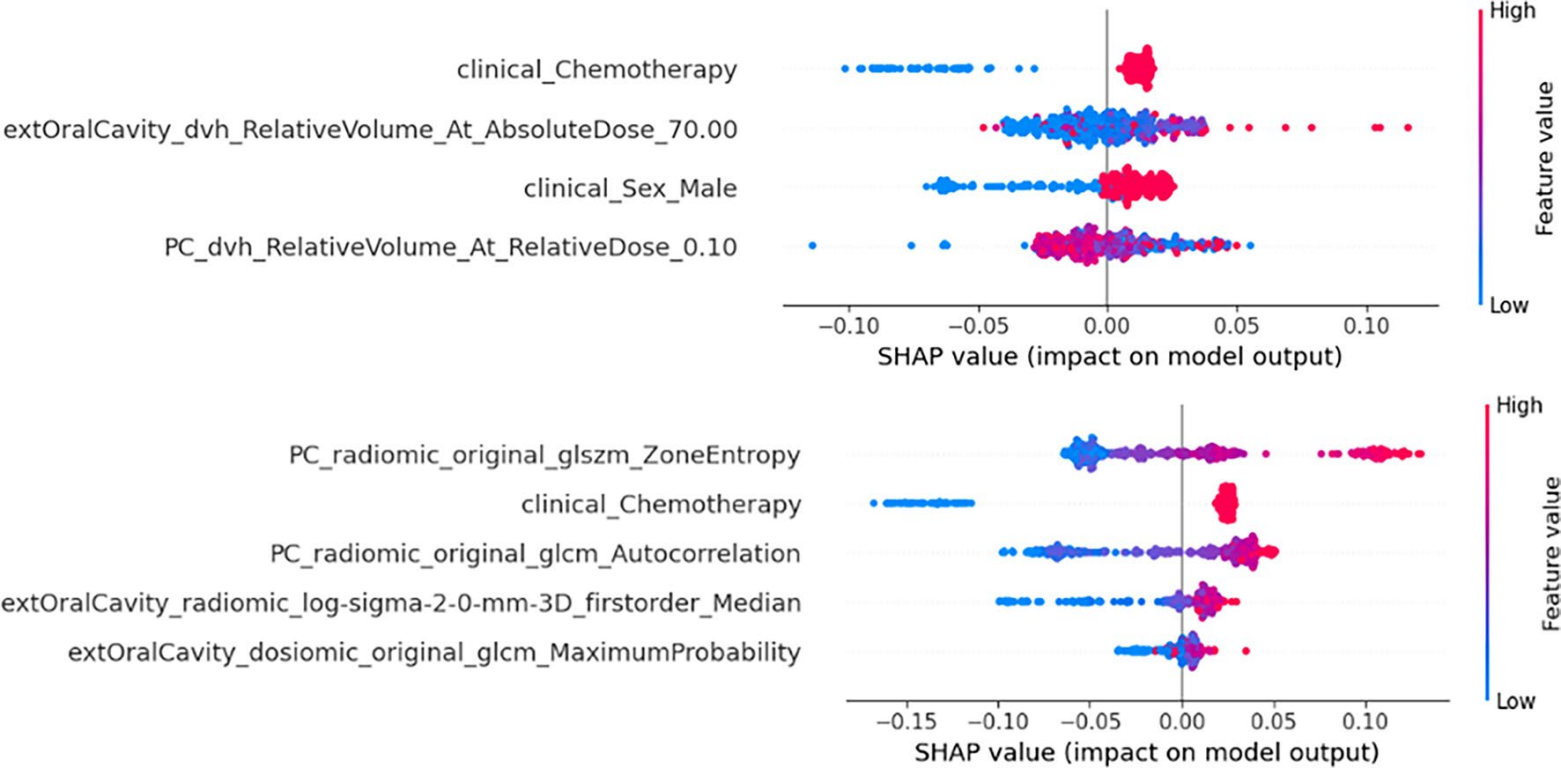
SHAP analysis of feature importance for the conventional model (top) and multi-omic model (bottom)

Figure 7 shows the SHAP feature importance for each model on the training dataset. The feature with greatest impact on the multi-omic model was the radiomic GLSZM zone entropy for the pharyngeal constrictor muscle. The plot indicates that higher heterogeneity in the texture in this volume resulted in a higher predicted probability for severe OM.

### Permutation feature importance

The impact of each model feature on model performance was also assessed using a model-agnostic permutation variable importance procedure [57]. The effect of removing each feature from the model was assessed by calculating the AUC on 1000 sets of bootstrapped samples after shuffling the values of the selected feature. The greater the impact of the feature, the larger the difference between the original AUC and the AUC after shuffling the feature.

**Fig. 8:**
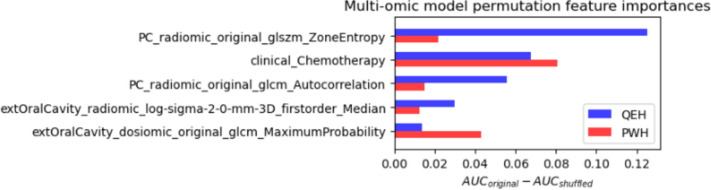
Permutation feature importances for multi-omic model

The results of the permutation feature importance assessment are shown in Fig. 8. Positive values indicate that shuffling the feature caused a decline in the model performance. The plot shows that all of the included features had a net positive impact on model performance, suggesting that removing any of these features would likely not improve model performance.

### Calibration

The machine learning models developed in this study were optimized for discrimination performance, which does not guarantee good calibration. Therefore, the final models were re-calibrated using the Scikit-learn CalibratedClassifierCV object. Prior to assessing the calibration and performing decision curve analysis, a dataset-specific logistic probability mapping was applied to the model. The feature coefficients of the underlying model were unchanged, and therefore the discrimination performance in AUC was also unchanged by this calibration.

Model calibration was assessed by plotting calibration curves and calculating the Brier score. For the calibration curves, the raw model predictions were binned into 5 quantiles, averaged and plotted against the ratio of positive cases in each bin. The slope and intercept of the resulting curves could then be compared to the ideally calibrated line. The Brier score ranged from 1, indicating a completely incorrect calibration, to 0, indicating a perfect calibration. Considering both the curve and the Brier score allowed for assessment of the overall calibration. Predicted probability bins were defined using quantiles rather than being uniformly distributed, in order to ensure an equal number of samples per bin and an equal significance of each point on the curve.

**Fig. 9:**
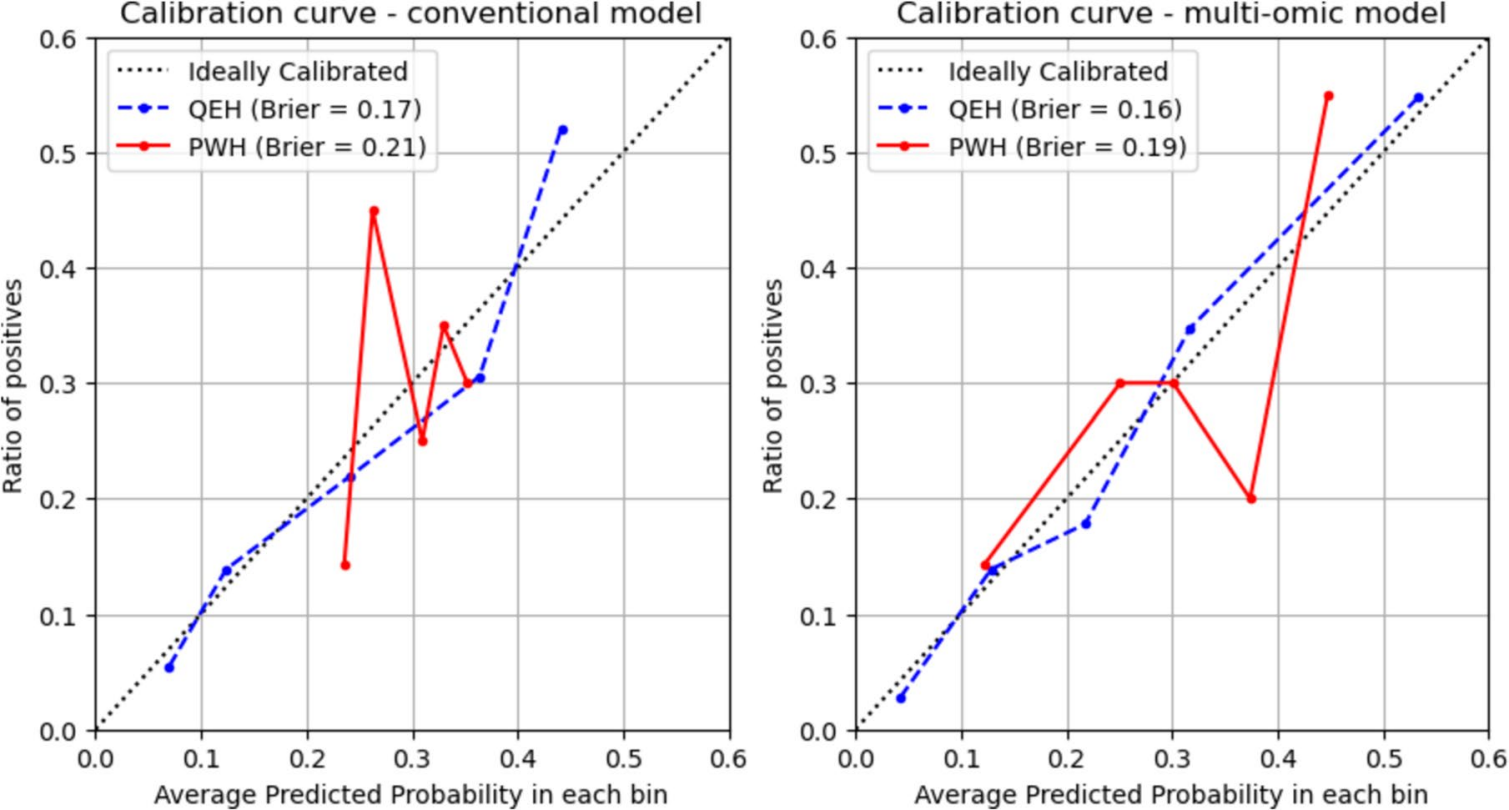
Calibration curves for the conventional model (left) and multi-omic model (right)

The calibration curves for the conventional (left) and multi-omic (right) models are shown in Fig. 9. For both models, the calibration on the development dataset was better than on the external validation set, as indicated by the lower Brier score and greater alignment of the calibration curve with the ideal calibration line. The multi-omic model exhibited a greater range in predicted probabilities and a lower Brier score than the conventional model, indicating superior calibration.

### Confusion matrix, sensitivity and specificity

The CLEAR guidelines recommend the inclusion of confusion matrices, which can illustrate the results of the prediction model for a given prediction threshold. An optimum prediction threshold was determined from the calibrated models using the Youden index, that is, the point which maximizes the sensitivity and specificity [58]. The resulting confusion matrices were calculated, along with the resulting sensitivity, specificity and overall accuracy. While the threshold can be adjusted depending on clinical requirements, these metrics serve as an additional means of comparison between models for a reasonable prediction threshold.

The confusion matrices for the conventional and multi-omic models calibrated on QEH and PWH are shown in Table 8. The sensitivity, specificity and accuracy for the multi-omic model were greater than those of the conventional model on the QEH dataset. On the PWH dataset, the conventional model resulted in a prediction which had a large number of false positives, while the multi-omic model prediction was less sensitive but more specific.

**Table 8:** Confusion matrix, sensitivity and specificity for conventional model and multi-omic model

	Prediction							
	Conventional model				Multi-omic model			
	Training (QEH)		External validation (PWH)		Training (QEH)		External validation (PWH)	
	OM G1-2	OM G3 +	OM G1-2	OM G3 +	OM G1-2	OM G3 +	OM G1-2	OM G3 +
OM G1-2	186	87	18	53	204	69	55	16
OM G3 +	30	60	3	27	27	63	16	14
Sensitivity	0.667		0.900		0.700		0.467	
Specificity	0.681		0.254		0.747		0.775	
Accuracy	0.678		0.446		0.736		0.683	

## Appendix I: CLEAR checklist

**Table:** 

Section	No	Item	Yes	No	N/A	Page
Title						
	1	Relevant title, specifying the radiomic methodology	☑	☐	☐	1
Abstract						
	2	Structured summary with relevant information	☑	☐	☐	2
Keywords						
	3	Relevant keywords for radiomics	☑	☐	☐	3
Introduction						
	4	Scientific or clinical background	☑	☐	☐	3
	5	Rationale for using a radiomic approach	☑	☐	☐	4
	6	Study objective(s)	☑	☐	☐	5
Method						
Study Design	7	Adherence to guidelines or checklists (e.g., CLEAR checklist)	☑	☐	☐	6
	8	Ethical details (e.g., approval, consent, data protection)	☑	☐	☐	7
	9	Sample size calculation	☑	☐	☐	8
	10	Study nature (e.g., retrospective, prospective)	☑	☐	☐	7
	11	Eligibility criteria	☑	☐	☐	7
	12	Flowchart for technical pipeline	☑	☐	☐	7
Data	13	Data source (e.g., private, public)	☑	☐	☐	7
	14	Data overlap	☐	☑	☐	
	15	Data split methodology	☑	☐	☐	8
	16	Imaging protocol (i.e., image acquisition and processing)	☑	☐	☐	8
	17	Definition of non-radiomic predictor variables	☑	☐	☐	8
	18	Definition of the reference standard (i.e., outcome variable)	☑	☐	☐	8
Segmentation	19	Segmentation strategy	☑	☐	☐	8
	20	Details of operators performing segmentation	☑	☐	☐	9
Pre-processing	21	Image pre-processing details	☑	☐	☐	20
	22	Resampling method and its parameters	☑	☐	☐	21
	23	Discretization method and its parameters	☑	☐	☐	21
	24	Image types (e.g., original, filtered, transformed)	☑	☐	☐	21
Feature extraction	25	Feature extraction method	☑	☐	☐	20
	26	Feature classes	☑	☐	☐	9, 20
	27	Number of features	☑	☐	☐	9
	28	Default configuration statement for remaining parameters	☐	☑	☐	20
Data preparation	29	Handling of missing data	☑	☐	☐	19
	30	Details of class imbalance	☑	☐	☐	12
	31	Details of segmentation reliability analysis	☑	☐	☐	21
	32	Feature scaling details (e.g., normalization, standardization)	☑	☐	☐	22
	33	Dimension reduction details	☑	☐	☐	22
Modeling	34	Algorithm details	☑	☐	☐	10,22
	35	Training and tuning details	☑	☐	☐	22
	36	Handling of confounders	☑	☐	☐	12
	37	Model selection strategy	☑	☐	☐	10
Evaluation	38	Testing technique (e.g., internal, external)	☑	☐	☐	10
	39	Performance metrics and rationale for choosing	☑	☐	☐	11
	40	Uncertainty evaluation and measures (e.g., confidence intervals)	☑	☐	☐	11, 27
	41	Statistical performance comparison (e.g., DeLong’s test)	☑	☐	☐	13
	42	Comparison with non-radiomic and combined methods	☑	☐	☐	12
	43	Interpretability and explainability methods	☑	☐	☐	11
Results						
	44	Baseline demographic and clinical characteristics	☑	☐	☐	12
	45	Flowchart for eligibility criteria	☑	☐	☐	8
	46	Feature statistics (e.g., reproducibility, feature selection)	☑	☐	☐	25
	47	Model performance evaluation	☑	☐	☐	13
	48	Comparison with non-radiomic and combined approaches	☑	☐	☐	13
Discussion						
	49	Overview of important findings	☑	☐	☐	15
	50	Previous works with differences from the current study	☑	☐	☐	15
	51	Practical implications	☑	☐	☐	15
	52	Strengths and limitations (e.g., bias and generalizability issues)	☑	☐	☐	17
Open Science						
Data availability	53	Sharing images along with segmentation data [n/e]	☐	☑	☐	–
	54	Sharing radiomic feature data	☐	☑	☐	–
Code availability	55	Sharing pre-processing scripts or settings	☐	☑	☐	–
	56	Sharing source code for modeling	☐	☑	☐	–
Model availability	57	Sharing final model files	☐	☑	☐	–
	58	Sharing a ready-to-use system [n/e]	☐	☑	☐	–

**Yes**, details provided; **No**, details not provided; **n/e**, not essential; **n/a**, not applicable.
